# *Wheat yellow mosaic virus* resistant line, ‘Kitami-94’, developed by introgression of two resistance genes from the cultivar ‘Madsen’

**DOI:** 10.1270/jsbbs.21101

**Published:** 2022-09-02

**Authors:** Takako Suzuki, Yasuhiro Yoshimura, Shizen Ohnishi, Hironobu Jinno, Tatsuya Sonoda, Masashi Kasuya, Chihiro Souma, Tetsuya Inoue, Masatomo Kurushima, Akira Sugawara, Shinji Maeno, Takao Komatsuda

**Affiliations:** 1 Hokkaido Research Organization Central Agricultural Experiment Station (AES), Naganuma, Yubari-gun, Hokkaido 069-1395, Japan; 2 Hokkaido Research Organization Kitami AES, Kunneppu, Tokoro-gun, Hokkaido 099-1496, Japan; 3 Hokkaido Research Organization Kamikawa AES, Pippu Kamikawa-gun, Hokkaido 078-0311, Japan; 4 Hokkaido Research Organization Tokachi AES, Memuro, Kasai-gun, Hokkaido 082-0081, Japan; 5 Institute of Crop Sciences, National Agriculture and Food Research Organization, Tsukuba, Ibaraki 305-8602, Japan; 6 Crop Research Institute, Shandong Academy of Agricultural Sciences, Jinan, Shandong 250100, China

**Keywords:** *Triticum aestivum* L., breeding, backcross, *Wheat yellow mosaic virus*, resistance genes

## Abstract

‘Kitahonami’ is a soft red winter wheat (*Triticum aestivum* L.) cultivar that has high yield, good agronomic performance and good quality characteristics. It currently accounts for 73% of the wheat cultivation area of Hokkaido the northern island in Japan and 42% of Japan’s overall wheat cultivation. However, this cultivar is susceptible to *Wheat yellow mosaic virus* (WYMV). WYMV has become widespread recently, with serious virus damage reported in Tokachi and Ohotsuku districts, which are the main wheat production areas in Hokkaido. Here, we report a new wheat breeding line ‘Kitami-94’, which was developed over four years by repeated backcrossing with ‘Kitahonami’ using DNA markers for WYMV resistance linked to the *Qym1* and *Qym2* from ‘Madsen’. Basic maps of *Qym1* and *Qym2* were created and used to confirm that ‘Kitami-94’ reliably carried the two resistance genes. ‘Kitami-94’ demonstrated WYMV resistance, and had agronomic traits and quality equivalent to ‘Kitahonami’ except for higher polyphenol oxidase activity and lower thousand grain weight. ‘Kitami-94’ may be useful for elucidating the mechanism of WYMV resistance in the background of ‘Kitahonami’, and for developing new cultivars.

## Introduction

‘Kitahonami’ is a leading wheat cultivar in Japan, with high yield and good agronomic performance. This cultivar is a soft red winter wheat with good milling and noodle-making quality, low ash content, and excellent flour color (Yanagisawa *et al.* 2007). Since 2006, this cultivar has been recommended by the government of Hokkaido, the northern island of Japan where 66% of Japanese wheat is produced. ‘Kitahonami’ is highly valued by farmers, millers, and processors, and was grown on about 73% of the wheat cultivation area in Hokkaido in 2019 (Anonymous 2021).

Unfortunately, ‘Kitahonami’ is susceptible to *Wheat yellow mosaic virus* (WYMV). WYMV was first described in Japan by Sawada (1927). Initially, damage was reported primarily in western Japan (Ikata and Kawai 1940), but WYMV appeared in Hokkaido in 1991 (Kusume *et al.* 1997). Since that time, it has become widespread in Hokkaido. The number of Hokkaido municipalities with WYMV-infested fields increased from 6 in 1994 to 57 in 2010 (Horita *et al.* 2011). In recent years the damage caused by WYMV has been serious in Tokachi and Ohotsuku districts, which are the main wheat production areas in Hokkaido and are widely planted with ‘Kitahonami’. New wheat cultivars are needed to resist this disease.

WYMV-resistant germplasm, and molecular markers linked to their resistance genes, have been developed. For example, ‘Madsen’ is a WYMV resistant cultivar developed in the USA (Allan *et al.* 1989). By combining two ‘Madsen’ alleles at the *Qym1* and *Qym2* QTLs, WYMV was completely controlled in virus nursery fields in Hokkaido (Liu *et al.* 2016, Suzuki *et al.* 2015). Although this research is still at the experimental level, the data has encouraged use of a DNA marker-assisted repeated backcrossing approach to breed WYMV resistant cultivars within a short timeframe. *Qym1* has been linked to *Xwmc041* in chromosome 2DL, and *Qym2* has been linked to *Xwmc754* in chromosome 3BS (Suzuki *et al.* 2015).

The initial aim of our research project was to generate a new wheat cultivar that is resistant to both WYMV and eye spot (*Pseudocercosparella herpotrichoides*). The BC_1_F_4_ line 4196-228 was originally selected for this dual purpose. However, due to the urgent need for a new cultivar with WYMV resistance, the focus of the work changed to WYMV resistance alone. The goals of the work reported here include creation of basic maps of *Qym1* and *Qym2* as single factor genes and development of near isogenic lines (NILs) of ‘Kitahonami’ using molecular markers closely linked to these QTLs, and evaluation of the WYMV resistance, grain yield, agronomic traits, and quality of grain and flour of the NILs, to develop a new line with WYMV resistance in addition to performance and quality equivalent to that of ‘Kitahonami’.

## Materials and Methods

### Plant materials

**Fig. 1.**
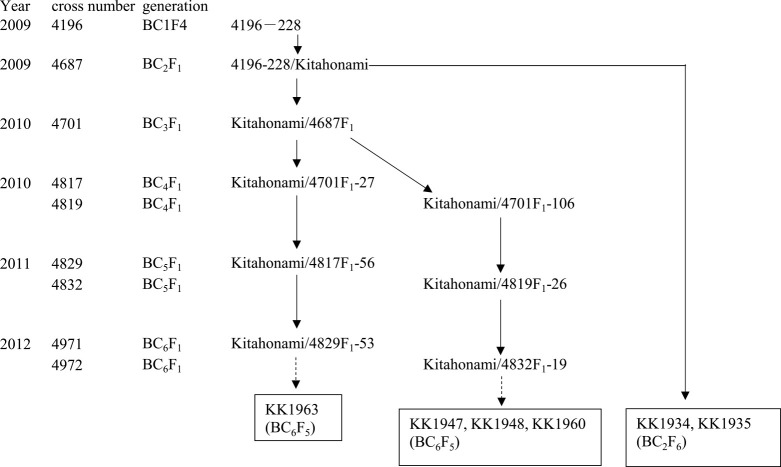
Breeding of ‘Kitahonami’ near-isogenic lines (NILs). NILs are shown in boxes.

Prior to the work reported here, back crossing had been performed with cross number 4196, using the recurrent parent ‘Kitami-81’ (subsequently named ‘Kitahonami’) and the WYMV resistance donor cultivar ‘Madsen’. Cross 4196 (BC_1_F_2_) was harvested without selection in 2007. A total of 690 plants in the BC_1_F_3_ generation were evaluated for stem length and maturity date, and 141 plants were selected by their spike and seed appearance to create breeding lines. The population was also advanced by selection of agronomic traits in the BC_1_F_2_ and BC_1_F_3_ generations. Ninety-two BC_1_F_4_ lines were genotyped using markers at *wmc041*, *wmc754* (Supplemental Table 1; Suzuki *et al.* 2015), and XustSSR2001-7DL (Groenewald *et al.* 2003). Line 4196-228, with ‘Madsen’ genotype at three markers was crossed with ‘Kitahonami’ in 2009, repeatedly backcrossed, and NILs were selected (Fig. 1).

### Marker assisted selection

Eight genetic markers linked to the resistance genes (Supplemental Table 1) were used to select plants. The marker, *ym115*, was converted from an AFLP fragment according to the methods of Suzuki *et al.* (2013), using resistant and susceptible recombinant inbred lines from the cross between the cultivar ‘Hokushin’ (WYMV susceptible; Yanagisawa *et al.* 2000) and ‘Madsen’. Analysis of *ym115* and *cfp059* was performed using Hotstar taq (QIAGEN, Hilden, Germany) and annealing temperature was 55°C. SSR markers were identified using Taq Gold DNA polymerase (Applied Biosystems, Foster City, CA, USA) and annealing temperature was 56°C. PCR products were analyzed with an ABI Prism 3500 Genetic Analyzer (Applied Biosystems) with GeneMapper software, as described by Suzuki *et al.* (2015) except that 2% agarose gel was used for *ym115* and *cfp059*. Individual plants that were heterozygous at all eight markers were selected in the BC_n_F_1_ generations, and individuals homozygous at four markers near the peak of the QTL were selected in the BC_n_F_2_ generations, to fix a QTL genotype. Selection in BC_n_F_3_ was based on agronomic trait scores being similar to those of ‘Kitahonami’.

### WYMV disease severity and infection rate

Disease severity was evaluated visually in the Date nursery and rated on a 0 to 4 scale (not infected to severely infected, respectively), as described by Takeuchi *et al.* (2010). Double-antibody sandwich enzyme-linked immunosorbent assay (ELISA; Clark 1981) was performed using polyclonal antibodies to WYMV (Ueda *et al.* 1998). Bulked samples of five plants per line and one leaf segment per plant were analyzed. Lines that were visually scored 0 and were WYMV negative in the ELISA test were classed as resistant; all other lines were classed as susceptible.

The WYMV resistance of ‘Kitami-94’ was further evaluated in WYMV-infested fields located in Date, Chitose, Sarabetsu, Tanno, and Kitami, Japan. Samples comprised of one leaf segment from each of 30 individual plants in a plot were collected in April of each year, and were analyzed by ELISA, as above.

### Agronomic traits

Agronomic performance was assessed by growing the breeding lines in multiple field locations in Japan, i.e., at the Chuo Agricultural Experiment Station (AES) in Naganuma, at Kamikawa AES in Pippu, at Tokachi AES in Memuro, at Kitami AES in Kunneppu, and in the WYMV-infested fields. All entries were sown in the middle of September. The plot design was randomized block with two replications, but ‘Kitami-94’ was tested in four replications at three AES and in six replications at Kitami AES. Cultivation details for the experimental field plots are listed in Supplemental Table 2. Agronomic traits including heading date, maturity date, stem length, number of spikes, grain yield, thousand grain weight, and test weight were assessed, either in the field or in the lab after harvest. Protein content of the harvested grain was measured using a near-infrared reflectance instrument (Infratec 1241, Foss, Denmark).

### Quality of flour and ‘udon’ noodles

Grains were milled using a Buhler test mill after conditioning the moisture level to 14.5% (w/w) overnight. Straight flour yield was calculated as the total recovered products. Flours were adjusted to 60% extraction in all subsequent tests. The ash content was determined using the rapid magnesium acetate method (American Association of Cereal Chemists 2000). The pasting color of the flours (6 g flour + 10 ml distilled water) was evaluated with a colorimeter (ZE 6000, Nippon Denshoku Industries, Tokyo, Japan). Amylose content was measured by starch-iodine reaction. Briefly, the absorbance at 600 nm of wheat flour samples (100 mg) was measured in an automated flow analyzer (AutoAnalyzer II, Bran Luebbe, Hamburg, Germany), using the method described by Juliano (1971). Udon (Japanese white salted noodles) were prepared and evaluated according to the method described by Toyokawa *et al.* (1989a, 1989b). Boiled noodles were scored by five trained panelists. The maximum score of 25 was given for color, 20 for surface appearance, 10 for appropriate firmness, 25 for elasticity, 10 for smoothness in the mouth, and 10 for a natural flavor.

### Polyphenol oxidase activity and genotyping of *PPO-D1*

Polyphenol oxidase (PPO) activity of the grain and the flour was measured using the dihydroxy-L-phenylalanine (L-DOPA) method of Anderson and Morris (2001), for grain, the L-DOPA method modified by Ito *et al.* (2008) for flour. Five grains were incubated with shaking at 20°C for 1 hour in 1.5 ml L-DOPA solution (10 mM L-DOPA, 50 mM 3-morpholinopropanesulfonic acid, pH 6.5) in a tube and PPO activities were determined by measuring absorbance of the supernatant at 475 nm with a spectrophotometer (U-2900, HITACHI Ltd., Tokyo, Japan). For flour, 0.2 g of flour and 4 ml of L-DOPA solution were placed in a flat-bottom test tube and shaken for 1 hour at room temperature, incubated at 20°C for 18 hours, stirred well, and measured with the colorimeter (ZE 6000, Nippon Denshoku Industries, Tokyo, Japan). For the genotyping of *PPO-D1*, we used *PPO29* marker described by He *et al.* (2007). Analysis of *PPO29* was performed using Hotstar taq (QIAGEN, Hilden, Germany) and annealing temperature was 55°C.

### Mapping of *Qym1* and *Qym2*

QTL analysis has located *Qym1* in an interval between *Xgwm539* and *Xgwm349* on the long arm of 2D, and *Qym2* in an interval between *Xbarc147* and *Xwmc623* on the short arm of chromosome 3B (Suzuki *et al.* 2015). In the current study, populations of F_2_:F_3_ progeny and four types of ‘Kitahonami’ and ‘Hokushin’ NILs were developed, in which *Qym1* and *Qym2* were homozygous for either the ‘Madsen’ or the recurrent parent genotype.

The *Qym1* locus was mapped using a segregating population derived from a ‘Kitahonami’ NIL. Thirty-six BC_6_F_2_ seeds derived from ‘Kitahonami’/4832F_1_-19 (Fig. 1) were sown in 2012, and three plants that were heterozygous at *Qym1* and homozygous for the ‘Madsen’ genotype at *Qym2* were selected to make a mapping population of *Qym1* using DNA markers (Supplemental Table 1). These plants, equivalent to the heterozygous F_1_ generation, were self-pollinated and F_2_ equivalent seeds were harvested in March 2014. Immediately 200 seeds were sown in the greenhouse to obtain F_3_ equivalent lines in August 2014. A total of 102 F_3_ equivalent lines that produced more than 60 seeds were sown in the WYMV-infested nursery in two replicates of 30 seeds each, and the WYMV resistance of the plants was evaluated in 2015. These 102 lines were genotyped by DNA markers flanking *Qym1*.

The *Qym2* locus was mapped using a segregating population derived from a ‘Hokushin’ NIL. Eighty BC_6_F_2_ seeds derived from a cross between Takikeimugi-3 (BC_5_ line generated by using ‘Hokushin’ as recurrent parent and ‘Madsen’ as resistance donor parent; Takeuchi *et al.* 2010) and ‘Hokushin’ were sown in 2010, and one plant that was homozygous at *Qym1* and heterozygous at *Qym2* was selected as the mapping population of *Qym2* using the markers described by Suzuki *et al.* (2015). This plant, equivalent to the heterozygous F_1_ generation, was self-pollinated and 262 F_2_ equivalent seeds were sown in 2012. One spike of each individual was bagged at heading date to prevent outcrossing, and the bagged spikes were harvested in 2013. A total of 104 F_3_ equivalent lines that yielded more than 40 seeds each were sown in two replicates of 20 seeds in the WYMV nursery, and the virus resistance of the plants was evaluated in 2014. These F_3_ lines were genotyped by DNA markers around *Qym2*. The polymorphic markers used for mapping were compared to web page data (https://wheat.pw.usda.Gov/ggpages/SSRclub/GeneticPhysical/) and to *cfp059* (Paux *et al.* 2008).

Three replications of the ‘Kitahonami’- and ‘Hokushin’-NILs were sown in 2013 and the virus infection rate of 20 individual plants was scored in 2014. Basic linkage maps were constructed using MAPMAKER/Exp v3.0b (Lander *et al.* 1987). Recombination frequencies were converted into map distances using Kosambi’s mapping function (Kosambi 1943).

## Results

### Line 4196-228 for resistance breeding

In the BC_1_F_4_ generation only a single line, 4196-228, was homozygous for the ‘Madsen’ genotype at *wmc041* (linked to *Qym1*) and *wmc754* (linked to *Qym2*) and heterozygous at XustSSR2001-7D. Among the 35 NILs that were grown in the WYMV nursery in 2010 and their WYMV infection rates scored, 4196-228 was judged to be resistant to infection by the virus. This line was homozygous for the ‘Madsen’ genotype at markers *wmc601* through *gwm349* flanking the *Qym1* locus, and *gwm389* (located between *barc147* and *gwm493*) through *wmc623* flanking the *Qym2* locus (marker locations as reported by Suzuki *et al.* 2015). Therefore line 4196-228 was selected for further backcrossing (Fig. 1).

### Agronomic traits and quality of NILs

Six NILs were evaluated for agronomic traits and quality from 2015 to 2017. The stem length in all NILs was 2 to 4-cm shorter (*P* < 0.05) than that of ‘Kitahonami’ grown in the same year, except for KK1935 which was not significantly different from ‘Kitahonami’ (Table 1). Thousand grain weight (TGW) of KK1947, KK1960, and KK1963 was 0.9, 3 and 2.4 g lighter, respectively, (*P* < 0.05) than that of ‘Kitahonami’. Heading date, maturity date, spike length, number of spikes, grain yield, test weight, and protein content of seeds of these NILs were not significantly different from the values for ‘Kitahonami’ (Table 1). The flour quality of these lines were very similar to each other (Supplemental Table 3). Line KK1947 had agronomic trait values and quality very similar to ‘Kitahonami’ and its TGW was heavier than that of KK1948 (Table 1). It was named ‘Kitami-94’, and was tested for certification as a recommended cultivar for Hokkaido. The new promising line ‘Kitami-94’ was studied extensively in the subsequent phases of the research reported here.

### Agronomic traits and quality of ‘Kitami-94’ grown in non-infested fields

Mean stem length of ‘Kitami-94’ was 3- and 2-cm shorter than that of ‘Kitahonami’ (*P* < 0.05) at the Tokachi AES and Kitami AES, respectively (Table 2). The TGW of ‘Kitami-94’ was lighter (*P* < 0.05) than that of ‘Kitahonami’ at all AES locations, and the test weight of ‘Kitami-94’ was lighter (*P* < 0.01) than that of ‘Kitahonami’ at three of the four AES locations. Mean seed protein content in ‘Kitami-94’ was 2 points lower (*P* < 0.05) than that of ‘Kitahonami’ at the Kitami AES only (Table 2). There were no significant differences between ‘Kitami-94’ and ‘Kitahonami’ in the other agronomic traits.

There were no significant differences between ‘Kitami-94’ and ‘Kitahonami’ in flour yield, grain ash content, flour protein content, flour color, or amylose content (Table 3). PPO activity was higher in ‘Kitami-94’ than in ‘Kitahonami’ in both grain (*P* < 0.05) and flour (*P* < 0.01) (Table 4). The *PPO-D1* genotype in ‘Kitami-94’ was the active type (*PPO-D1b*), which was the same as in ‘Madsen’. Although more gray spots were observed on the uncooked noodle sheet in ‘Kitami-94’ than in ‘Kitahonami’ (data not presented), the cooked noodle scores were not significantly different from each other (Table 3).

### Agronomic traits of ‘Kitami-94’ grown in WYMV-infested fields

The WYMV infection rates in ‘Kitahonami’ were much higher than in ‘Kitami-94’ in all WYMV-infested fields (Table 5). The heading dates and maturity dates of ‘Kitahonami’ were almost always 3 days later than those of ‘Kitami-94’. The mean stem length of ‘Kitahonami’ was shorter than that of ‘Kitami-94’ in four of the five fields (Table 5). Although these differences were not significant, the trend likely reflects the effects of the WYMV virus infection.

Test weight and TGW of ‘Kitami-94’ were not significantly different from those of ‘Kitahonami’ (Table 5). Importantly, however, the mean grain yield of ‘Kitahonami’ was significantly lower (*P* < 0.01) than that of ‘Kitami-94’ when both were grown in WYMV-infested fields, with differences ranging from 14% to 33% (Table 5). Also, the mean protein content of ‘Kitahonami’ was 0.4 points higher (*P* < 0.05) than that of ‘Kitami-94’, likely due to the reduced yields.

### Effect of single resistance genes on WYMV infection

All tested NILs, including ‘Kitami-94’, that inherited both *Qym1* and *Qym2* QTLs from ‘Madsen’ had almost complete resistance to WYMV (Tables 1, 5). With regard to the individual effect of each QTL, the WYMV infection rates were very similar regardless of the broader genetic background of the NIL. The infection rates of NILs with ‘Madsen’ alleles at both the *Qym1* and *Qym2* loci (*Qym1.m* and *Qym2.m*, respectively) were 0% (Table 6). Infection rates of NILs with only *Qym1.m* were *ca.* 45%, whereas in NILs with only *Qym2.m* the infection rates were *ca.* 30%. In NILs with no ‘Madsen’ alleles the infection rates were *ca.* 100% (Table 6).

### Mapping of *Qym1* and *Qym2*

**Fig. 2.**
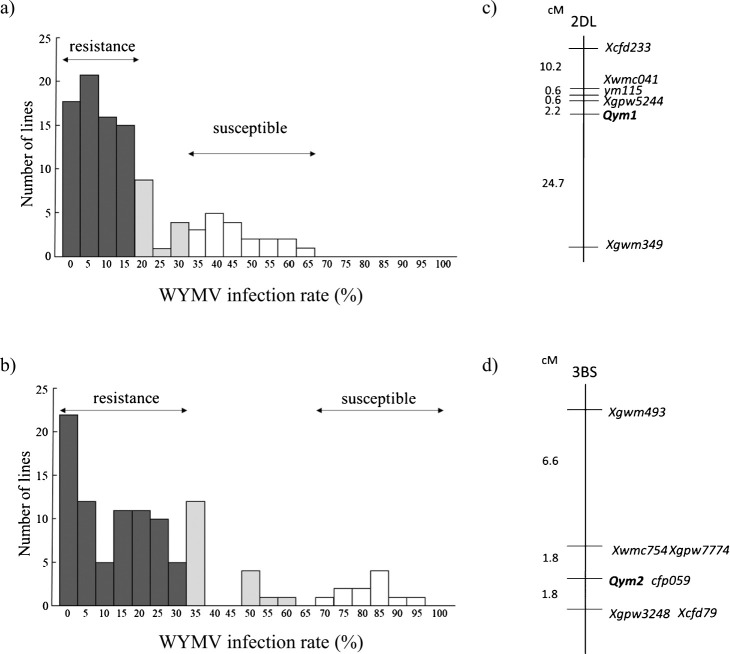
Frequency distributions of WYMV infection rate, and basic maps of *Qym1* and *Qym2*. a) F_2:3_ population segregating for *Qym1* while fixed for homozygous ‘Madsen’ genotype at *Qym2*; b) F_2:3_ population segregating for *Qym2* while fixed for homozygous ‘Madsen’ genotype at *Qym1*; c) linkage map of *Qym1*; d) linkage map of *Qym2*. Dark-shaded bars represent families judged to be resistant to WYMV; pale-shaded bars represent families with intermediate infection rates; unshaded bars represent families judged to be susceptible to WYMV. Families with intermediate infection rates were excluded from the gene mapping.

In the population of F_2_:F_3_ progeny (produced by selfing F_2_ equivalent plants), developed from the cross between ‘Kitahonami’ and 4832F_1_-19 (Fig. 1), the plants with more than 35% infection rate were considered to be *Qym1.k/Qym1.k* (i.e., homozygous ‘Kitahonami’), whereas those with infection rates below 15% were presumed to be either *Qym1.m/Qym1.m* or *Qym1.m/Qym1.k* (Fig. 2a). Marker analysis and phenotype analysis of 102 F_3_ equivalent progeny, indicated that the virus resistance was inherited in a monogenic manner, and that *Qym1* mapped at 2.2 cM distal to *Xgpw5244* (Fig. 2c).

In the population of F_2_:F_3_ progeny developed from the cross between ‘Takikeimugi-3’ and ‘Hokushin’, the plants with more than 70% infection rate were considered to be homozygous ‘Hokushin’ (*Qym2.h/Qym2.h*), and those with less than 35% infection rate were regarded as either homozygous *Qym2.m/Qym2.m* or heterozygous *Qym2.m/Qym2.h* (Fig. 2b). Marker analysis and phenotype analysis of 104 F_3_ equivalent progeny, indicated that the virus resistance was again inherited in a monogenic manner, and that *Qym2* was located in an interval of 3.6 cM between *Xwmc754* and *Xcfd79* and mapped to the same position as *cfp059* (Fig. 2d).

## Discussion

‘Kitami-94’ was bred from the original line 4196-228 over a period of four years. Basic mapping indicated that 4196-228 and all the resulting NILs reliably carried the two resistance genes, at *Qym1* and *Qym2*. This study confirmed single resistance effects of both *Qym1.m* and *Qym2.m* in the advanced backcross lines. It also found that *Qym2.m* reduced the WYMV infection rate more strongly than did *Qym1.m*.

‘Kitami-94’ developed in this study was highly resistant to WYMV but was otherwise similar to ‘Kitahonami’ in agronomic traits. The yield reduction observed in ‘Kitahonami’ grown in the WYMV-infested field was not observed in ‘Kitami-94’. The heading and maturity dates of WYMV-infested ‘Kitahonami’ were both later than those of ‘Kitami-94’. In the general production fields where ‘Kitahonami’ is unevenly infected by WYMV, the disease not only reduces yield but also make harvesting problematic due to the uneven maturity. In the non-infested fields, ‘Kitami-94’ had the same yield as ‘Kitahonami’, but had slightly higher spike number and smaller grain size (lower TGW) than ‘Kitahonami’. A gene increasing the spike number may have been responsible for the smaller grain size. For example, Suzuki *et al.* (2012) found that cultivar ‘Sumai3’ had a Fusarium head blight resistance QTL near *Xgwm539* on 2DL, and that this QTL was significantly correlated with decreased TGW. Interestingly, if the gene responsible for grain size on 2DL in ‘Madsen’ is identical to the ‘Sumai3’ gene reported by Suzuki *et al.* (2012), this identification of a gene responsible for grain weight near *Qym1* may facilitate wheat breeding for resistance to other diseases.

Although ‘Kitahonami’ is a high yielding cultivar, it sometimes produces narrow grains (Sakuma *et al.* 2019), particularly under unfavorable weather conditions. The TGW of ‘Kitami-94’ was typically lower than that of ‘Kitahonami’, which may be attributable to a grain size gene linked to *Qym1*. This characteristic might cause a problem that more thin grains are produced under unfavorable weather conditions.

The WYMV infection rates of ‘Kitahonami’ in the Chitose and Sarabetsu WYMV-infested fields were lower than was observed in the other WYMV-infested fields, however the yield reduction of ‘Kitahonami’ grown in these two fields was actually more severe than observed in the other WYMV-infested fields. The reason for this is not clear. Other yield-reducing diseases may have been present in these two fields, the yields of ‘Kitami-94’ grown there did not appear to be reduced. Perhaps there is another resistance gene near *Qym1* or *Qym2* that prevented yield loss in those fields, but considerable additional study would be needed to investigate this possibility.

The grain quality of ‘Kitami-94’ was very similar to that of ‘Kitahonami’, with the exception of PPO activity. ‘Kitami-94’ had the same active type of *PPO-D1b* as ‘Madsen’. ‘Kitami-94’, with highly active PPO, had more gray spots on the uncooked noodle sheet than ‘Kitahonami’. However, PPO activity of this sort is not a problem in making Japanese ‘*udon*’ noodles, because the noodles are boiled immediately after preparation. Thus the boiled ‘*udon*’ noodles of ‘Kitahonami’ and ‘Kitami-94’ were not visibly different. Importantly, however, wheat flour is also used to make various other products such as Chinese noodles and sweets and so on, gray spots might reduce the value of the product for example ramen and dumpling skins. The NILs and ‘Kitami-94’ had slightly shorter stem length than those of ‘Kitahonami’. Genes with a strong effect on plant height, such as *Rht-B1* on 4B and *Rht-D1*on 4D (Ellis *et al.* 2002, Gale and Law 1977), *Rht8* on 2DS (Korzun *et al.* 1998) and many others with minor effects have been reported. ‘Madsen’ may have a genetic factor, perhaps on 2DL or 3BS, that slightly shortens stem length. However, the effect on the NILs in this study was small, and did not appear to have a strong impact on other agronomic traits.

In their study of WYMV resistant NILs derived from ‘Madsen’, Kasuya *et al.* (2017) found that the lines in which the 2DL genotype is ‘Madsen’ have relatively lighter TGW and grain test weight. Zhang *et al.* (2015) reported that the QTLs related to TGW, grain width, and ratio of grain length to grain width are located near *Xwmc041* in a mutant generated by exposure to ethyl methanesulfonate. Based on this information, lines with TGW similar to that of ‘Kitahonami’ were selected in the work reported here, to break the linkage between resistance genes and small grain size. Despite this effort, the TGW of all of the selected lines in the current study was less than that of ‘Kitahonami’. This implies that it is difficult to break the linkage between WYMV resistance and TGW by normal selection, and it may be necessary to search for recombinant individuals using a much larger population. However, if the resistance gene can be isolated using the basic map of *Qym1* developed in this study, it may be possible to efficiently produce lines that eliminate the linkage with undesirable traits mentioned above.

It is likely that the ‘Kitami-94’ line will be useful for basic research to elucidate the mechanisms of PPO activity and resistance to WYMV. Kobayashi *et al.* (2020) produced lines with broken linkage between *Ppo-D1b* and WYMV resistance, using 960 plants from recurrent back cross progenies. With the help of this report we have begun to break the linkage of undesirable traits with *Qym1* on 2DL in ‘Kitami-94’. Future development of new lines without linkage drag could become a powerful breeding tool for pyramiding resistance genes. Recently we started wheat breeding combining *Qym1* and *Qym2* with a new WYMV resistance gene at *Qym4* derived from OW104 (Yamashita *et al.* 2020) in order to breed varieties with stronger resistance to WYMV. These approaches may help breeders respond to the ongoing changes in viral strains and climate.

The WYMV response alleles at *Qym1* and *Qym2* present in cvs ‘Madsen’ and ‘Hokushin’ are denoted *Ym1*/*Ym2* and *ym1*/*ym2*, respectively, according to recommendation rule for gene symbolization in wheat (McIntosh *et al.* 2013).

## Author Contribution Statement

YY, HJ, SO, TS, and TS produced experimental materials. TS, MK, CS, TI, MK, AS, and TS performed the experiments. TS and TK designed the experiments to evaluate resistance genes and basic maps. TS and TK analyzed the data and wrote the manuscript.

## Supplementary Material

Supplemental Tables

## Figures and Tables

**Table 1.** Agronomic traits of several ‘Kitahonami’ near-isogenic lines (NILs)

Year	Name	WYMV resistance (in Date)	Genreration	Heading date	Maturity date	Stem length (cm)	Spike length (cm)	Number of spikes (per m^2^)	Grain yield (kg ha^–1^)	1000-grain weight (g)	Test weight (g)	Protein content of seeds (%)
2015	KK1934	Resistant	BC_2_F_6_	6.01	7.22	81**	8.6	724	9374	41.3	828	9.3
	KK1935	Resistant	BC_2_F_6_	6.01	7.22	86	8.5	759	9591	41.9	833	9.3
	Kitahonami	Susceptible		6.01	7.22	85	8.7	627	9253	42.3	848	9.3
2016	KK1947	Resistant	BC_6_F_4_	6.01	7.25	84*	8.8	815	7494	40.7*	819	10.9
	KK1948	Resistant	BC_6_F_4_	6.02	7.25	83*	8.9	732	7518	39.8	823	11.2
	Kitahonami	Susceptible		6.01	7.25	86	8.6	811	7255	41.6	828	10.8
2017	KK1960	Resistant	BC_6_F_5_	6.05	7.20	81*	8.8	582	7478	37.9*	832	10.3
	KK1963	Resistant	BC_6_F_5_	6.05	7.20	81*	8.8	603	7415	38.5*	834	10.4
	Kitahonami	Susceptible		6.04	7.20	84	8.8	583	7248	40.9	845	10.3
Mean	NILs			6.02	7.22	83	8.7	703	8145	40.0	828.3	10.2
	Kitahonami			6.02	7.22	85	8.7	674	7918	41.6	840.3	10.1

*,** Significantly different from ‘Kitahonami’ at the 5% and 1% levels, respectively (Student’s *t*-test).

**Table 2.** Agronomic traits of ‘Kitami-94’ grown in several WYMV non-infested fields

Test field	Line and cultivar name	Heading date	Maturity date	Stem length (cm)		Spike length (cm)	Number of spikes (per m^2^)	Grain yield (kg ha^–1^)	1000-grain weight (g)		Test weight (g)		Seeds protein (%)	
Chuo AES	Kitami-94	6.01	7.18	85		9.2	806	8336	36.6	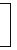 **	823	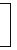 **	9.4	
	Kitahonami	6.01	7.17	86		9.2	793	8183	38.8		830		9.6	
Kamikawa AES	Kitami-94	6.04	7.18	74		8.6	458	5545	39.3	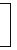 *	829	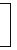 **	9.5	
	Kitahonami	6.04	7.18	74		8.7	460	5568	41.3		834		9.5	
Tokachi AES	Kitami-94	6.01	7.23	78	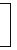 **	8.9	713	6435	40.0	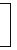 **	849	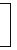 **	11.1	
	Kitahonami	6.02	7.23	81		8.9	665	6484	42.2		859		11.2	
Kitami AES	Kitami-94	6.06	7.26	78	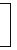 **	8.6	717	8866	40.4	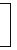 *	839		9.8	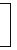 *
	Kitahonami	6.05	7.26	80		8.6	666	8691	42.3		844		10.0	
Mean of 4 AES	Kitami-94	6.02	7.21	79		8.8	674	7295	39.1		835		9.9	
	Kitahonami	6.03	7.21	80		8.9	646	7232	41.1		842		10.1	

Data are means of the results from 2017–2020. *,** Significantly different at the 5% and 1% levels, respectively (Student’s *t*-test).

**Table 3.** Flour and noodle quality of ‘Kitami-94’ and ‘Kitahonami’ grown in WYMV non-infested fields

Year	Name	Flour yield (%)	Grain ash (%)	Flour protein (%)	Pasting color of the flour			Amylose content (%)	Noodle scoring
					L*	a*	b*		
2017	Kitami-94	73.6	1.25	8.7	87.92	–0.35	15.79	22.4	69.9
	Kitahonami	74.8	1.24	8.7	87.70	–0.28	15.65	21.9	70.0
2018	Kitami-94	74.6	1.27	9.5	87.37	–0.04	16.89	21.8	69.8
	Kitahonami	73.8	1.25	9.7	87.79	–0.19	16.46	21.9	70.0
2019	Kitami-94	73.2	1.23	8.4	87.67	–0.07	16.89	20.5	70.2
	Kitahonami	73.2	1.27	8.8	87.62	–0.01	16.69	20.7	70.0
2020	Kitami-94	73.1	1.26	8.3	87.75	–0.15	16.63	20.9	69.9
	Kitahonami	72.4	1.22	8.4	87.89	–0.27	16.63	21.3	70.0
Mean	Kitami-94	73.8	1.25	8.7	87.68	–0.15	16.55	21.4	70.0
	Kitahonami	73.9	1.25	8.9	87.75	–0.19	16.36	21.5	70.0

**Table 4.** Flour properties and polyphenol oxidase (PPO) activity and *PPO-D1* allele

Sample	Flour ash content	Flour protein content	Grain PPO*^a^* (475 nm)		Flour PPO*^b^* (L*)		Genotype by *PPO29*
Kitahonami	0.37	9.7	0.229	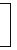 *	59.26	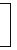 **	*PPO-D1a*
Kitami-94	0.40	9.5	0.325		56.18		*PPO-D1b*
Madsen*^c^*	–	–	–		–		*PPO-D1b*

Flour properties and PPO data are in 2017. *,** Significantly different from ‘Kitahonami’ at the 5% and 1% levels, respectively (Student’s *t-*test). *^a^* Grain PPO activity was determined by measuring absorbance of the supernatant at 475 nm with a spectrophotometer. *^b^* Flour PPO was measured by colorimeter using the dihydroxy-L-phenylalanine method. *^c^* For Madsen, only *PPO-D1* allele was tested.

**Table 5.** Agronomic traits of ‘Kitami-94’ and ‘Kitahonami’ grown in several WYMV-infested fields

Test field	Line and cultiver name	WYMV infection %	Heading date	Maturity date	Stem length (cm)	Spike length (cm)	Number of spikes (per m^2^)	Grain yield (kg ha^–1^)		1000-grain weight (g)	Test weight (g)	Seeds protein (%)	
Date	Kitami-94	0.9	5/29	7/19	78	9.2	839	7880		37.5	802	9.9	
	Kitahonami	99.2	6/1	7/20	73	8.4	694	6320		36.3	804	9.7	
Chitose	Kitami-94	0.0	–	–	79	9.1	745	8010		37.7	812	10.6	
	Kitahonami	34.5	–	–	80	9.5	714	5385		37.4	802	10.9	
Sarabetsu	Kitami-94	3.1	6/3	7/25	74	8.4	750	6820		39.5	855	11.7	
	Kitahonami	65.0	6/7	7/28	73	8.3	723	4810		35.7	846	12.4	
Tanno	Kitami-94	5.9	6/4	7/23	74	8.8	680	8525		39.1	826	10.4	
	Kitahonami	90.0	6/8	7/26	68	8.4	673	7185		38.7	819	10.9	
Kitami	Kitami-94	1.7	5/31	7/21	74	8.9	718	8330		39.0	825	10.8	
	Kitahonami	93.2	6/4	7/25	72	8.7	683	7195		39.8	834	11.8	
Mean of 5 area	Kitami-94	2.3	6/2	7/22	76	8.8	746	7913	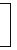 **	38.5	824	10.7	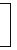 *
	Kitahonami	76.4	6/5	7/25	73	8.6	697	6179		37.6	821	11.1	

Data are means of results from 2018–2019 for the Date, Sarabetsu, Tanno, and Kitami fields, and from 2019–2020 for Chitose. *,** Significantly different at the 5% and 1% levels, respectively (Student’s *t*-test).

**Table 6.** WYMV infection rate by genetic background of NILs grown in a WYMV-infested field

a) ‘Hokushin’				b) ‘Kitahonami’		
Locus*^a^*		WYMV infection %		Locus*^b^*		WYMV infection %
*Qym1*	*Qym2*			*Qym1*	*Qym2*	
Madsen	Madsen	0.0		Madsen	Madsen	0.0
Madsen	Hokushin	46.7		Madsen	Kitahonami	45.0
Hokushin	Madsen	30.0		Kitahonami	Madsen	28.3
Hokushin	Hokushin	98.3		Kitahonami	Kitahonami	100.0

*^a^* Homozygous for *wmc041* and *ym115* in *Qym1* and *gwm493*, *wmc754*, *cfd79* and *gpw3248* in *Qym2* region. *^b^* Homozygous for *wmc041*, *ym115* and *gwm349* in *Qym1* and *gwm389*, *gwm493*, *wmc754*, *cfp1844* and *wmc623* in *Qym2* region.
