# Supplementary material for: Wheat yellow mosaic virus resistant line, ‘Kitami-94’, developed by introgression of two resistance genes from the cultivar ‘Madsen’
Source: Breed Sci. 2022 Sep 2;72(4):297–305. doi: 10.1270/jsbbs.21101 (PMC9868331; doi:10.1270/jsbbs.21101)
Supplement: Supplementary file 1 — Supplemental Tables [file 72_297_s1.pdf]

Supplemental Table 1. Primers used in PCR testing for selection among backcross progeny.

Marker	Locus	Primer	Sequence (5′ to 3′) [a]	Fragment size (bp) [b]	Notes
wmc601	*Xwmc601* (2D)	wmc601F-m13	cacgacgttgtaaaacgacACAGAGGCATATGCAAAGGAGG	Madsen 234	Near the end of QTL
		wmc601R	CTTGTCTCTTTATCGAGGGTGG	Kitahonami 249	
ym115	*ym115* (2D)	ym115-15	TGGAATGTGATGAGGCTC	Madsen 650	Near the peak of QTL
		ym115-26	CCTGTCTCACTCGTATGAATG	Kitahonami 360	
		ym115-28	ATACCAGCTCATGTGTTTAGTAC		
wmc041	*Xwmc041* (2D)	wmc041F-m13	cacgacgttgtaaaacgacTCCCTCTTCCAAGCGCGGATAG	Madsen 170	Near the peak of QTL
		wmc041R	GGAGGAAGATCTCCCGGAGCAG	Kitahonami 168 + 178	
gwm349	*Xgwm349* (2D)	gwm349F-m13	cacgacgttgtaaaacgacGGCTTCCAGAAAACAACAGG	Madsen 257	Near the end of QTL
		gwm349R	ATCGGTGCGTACCATCCTAC	Kitahonami 247	
gwm389	*Xgwm389* (3B)	gwm389F-m13	cacgacgttgtaaaacgacATCATGTCGATCTCCTTGACG	Madsen 138	Near the end of QTL
		gwm389R	TGCCATGCACATTAGCAGAT	Kitahonami 156	
wmc754	*Xwmc754.1* (3B)	wmc754F-m13	cacgacgttgtaaaacgacATCCACATGAACCTCAACTTATGG	Madsen 198	Near the peak of QTL
		wmc754R	GGCATTGTTGTTGTACTGCAGTC	Kitahonami 154	
cfp059	*cfp059* (3B)	cfp059F	CGACACAAGGAGAGGCAAAG	Madsen 220	Near the peak of QTL
		cfp059R	AAATCAACCAGACCACCATTGC	Kitahonami null	
wmc623	*Xwmc623* (3B)	wmc623F-m13	cacgacgttgtaaaacgacACGATTGGCCACAGAGGAG	Madsen 208	Near the end of QTL
		wmc623R	CAGTGACCAATAGTGGAGGTCA	Kitahonami null	

[a] The sequence 'cacgacgttgtaaaacgac' is the M13 tail.

[b] Fragment size differs from the actual size due to the analytical method used.

Supplemental Table 2. Overview of production data. Plots were arranged in randomized blocks at each location.

Location	Plots (m^2)	Rows space (m)	replications	seeding amount (seeds per m^2)	Fertilization amount at sowing (kg ha^{-1}) N	P_2O_5	K_2O	Additional fertilizer (kg ha^{-1}) N
Chuo AES	9.6	0.2	4	200	40	125	50	100
Kamikawa AES	9.6	0.3	4	255	40	100	60	100
Tokachi AES	9.6	0.3	4	255	40	160	96	120
Kitami AES	5.4	0.2	6	255	57	175	70	100
Date	12	0.3	2	255	72	90	72	40
Chitose	12	0.3	2	255	40	125	50	100
Sarabetsu	9.6	0.3	2	200	40	110	45	155
Tanno	12	0.3	2	200	40	150	75	126
Kitami	12	0.3	2	200	64	160	64	137

AES: Agricultural Experiment Station

Supplemental Table 3. Flour and noodle quality of several ‘Kitahonami’ near-isogenic lines (NILs).

Year	Name	Flour yield (%)	Grain ash (%)	Flour protein (%)	Pasting color of the flour L*	Pasting color of the flour a*	Pasting color of the flour b*	Amylose content (%)	Noodle scoring
2015	KK1934	71.9	1.32	8.7	88.13	-0.41	16.45	23.6	70.1
	KK1935	72.1	1.28	8.9	87.70	-0.28	16.88	22.6	69.8
	Kitahonami	71.8	1.25	9.6	87.88	-0.31	15.67	23.1	70.0
2016	KK1947	72.3	1.18	8.2	87.77	-0.47	17.39	22.1	70.3
	KK1948	71.9	1.2	8.3	87.84	-0.55	17.80	21.9	70.8
	Kitahonami	71.6	1.18	8.5	87.95	-0.58	16.71	21.6	70.0
2017	KK1960	75	1.28	9.1	87.52	-0.36	17.47	22.0	70.0
	KK1963	73.6	1.35	8.9	88.02	-0.47	16.43	21.7	70.2
	Kitahonami	74.3	1.27	8.7	87.65	-0.22	15.65	22.7	70.0
Mean	NILs	72.8	1.27	8.7	87.86	-0.42	17.07	22.3	70.2
	Kitahonami	72.6	1.23	8.9	87.83	-0.37	16.01	22.2	70.0
